# Supplementary figures and images for: COP9 signalosome is an essential and druggable parasite target that regulates protein degradation
Source: PLoS Pathog. 2020 Sep 22;16(9):e1008952. doi: 10.1371/journal.ppat.1008952 (PMC7531848; doi:10.1371/journal.ppat.1008952)

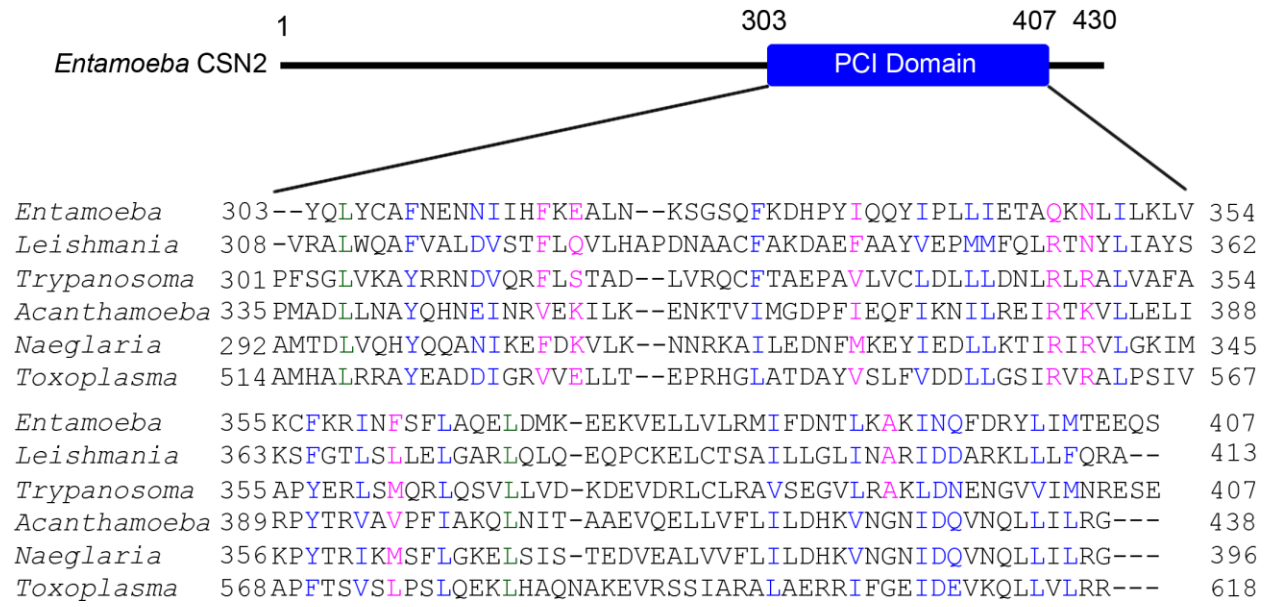

Supplement: S1 Fig — Entamoeba histolytica (EHI_174890A), Leishmania donovani (LdCL250028900-t42), Trypanosoma cruzi (Tb427.03.2320-t26), Acanthamoeba castellanii (ACAI_288500), Naegleria fowleri (Mrna1_nf0005120), Toxoplasma gondii (TGGT1_236220). Identical (green), conserved (blue), semi-conserved (pink), and non-conserved residues (black). (PDF) [file ppat.1008952.s001.pdf]

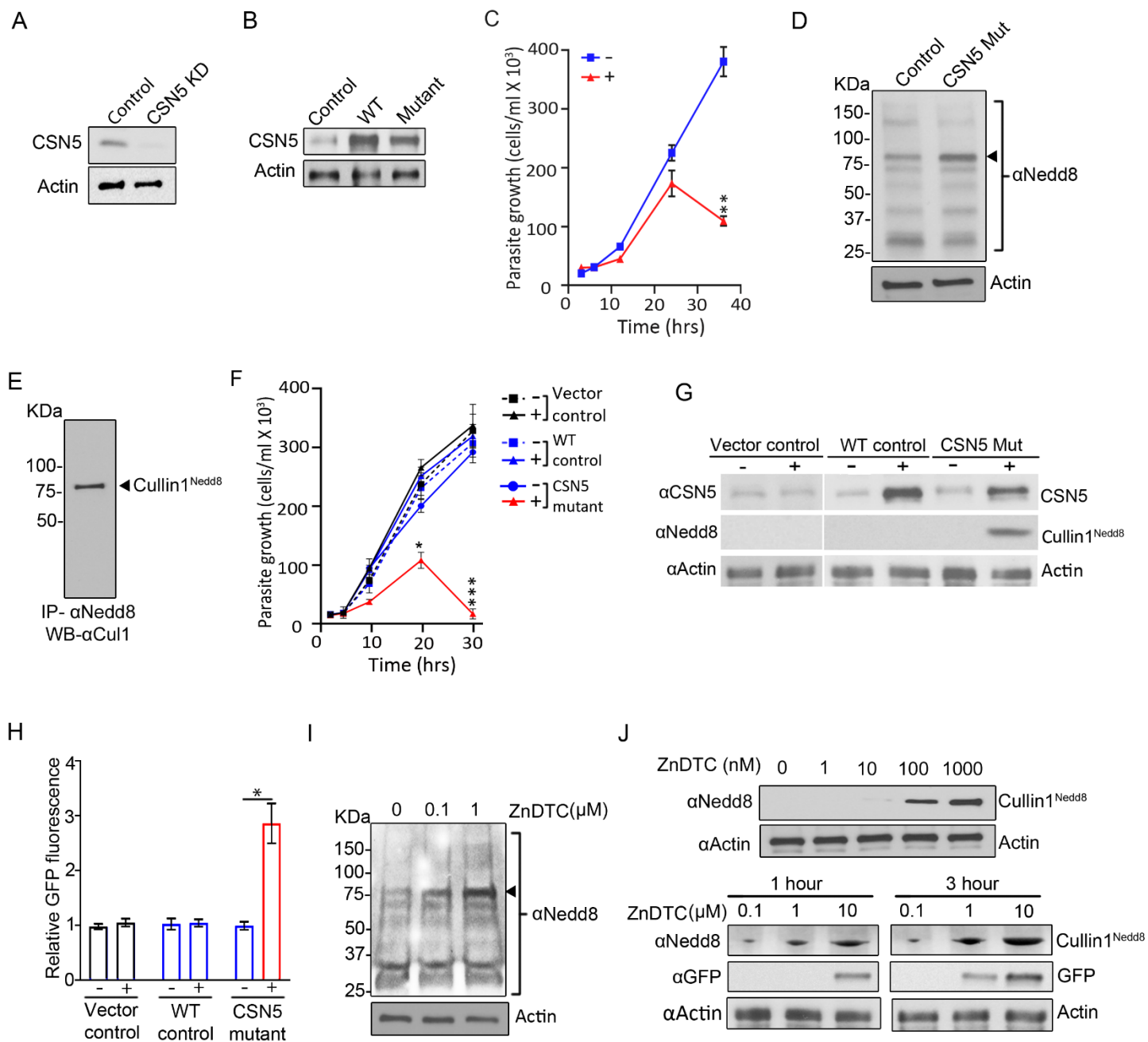

Supplement: S3 Fig — (A) Reduced CSN5 protein expression in knockdown cells analyzed by immunoblotting. (B) Overexpression of WT and mutant (D147N) CSN5 proteins. (C) Cell proliferation assay showing the effect of CSN5 knockdown on E. histolytica viability. Cells with tetracycline-inducible CSN5 knockdown construct with (+) and without (-) tetracycline. (D) Accumulation of neddylated proteins in catalytically inactive CSN5 mutant. (E) Immunoblot analysis of neddylated cullin1. (F) Dominant negative effect of the catalytically inactive CSN5 mutant. Cell proliferation assay showing reduced viability in cells overexpressing CSN5 mutant compared to WT CSN5 overexpression and vector controls with (+) and without (-) tetracycline. (G) Immunoblot analysis of CSN5 and neddylated Cullin1 expression in cells with vector, CSN5 WT or mutant construct with (+) and without (-) tetracycline. Actin used as loading control. (H) CSN5 mutant results in GFP accumulation. Fluorometric assay of GFP accumulation, presence (+) or absence (-) of tetracycline. *P < .05, ** P < 0.01, *** P < 0.001, two-tailed t test. (I) Accumulation of neddylated proteins in ZnDTC treated cells. (J) Dose-dependent inhibitory effect of ZnDTC treatment on the endogenous deneddylation of cullin1 (12 h). ZnDTC inhibitory effects at earlier time points. Actin used as loading control. (PDF) [file ppat.1008952.s003.pdf]

A

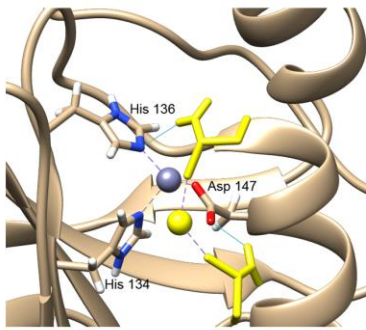

B

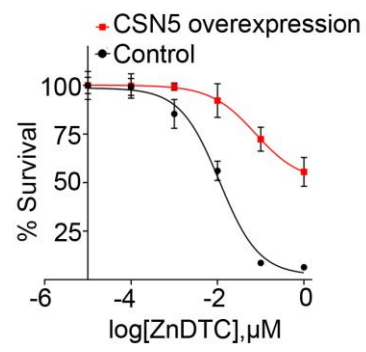

C

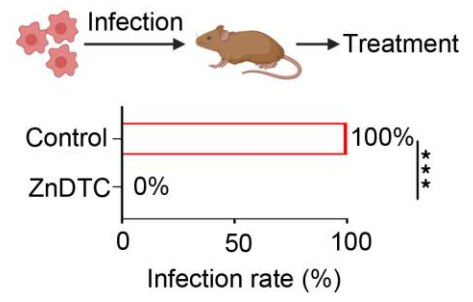

Supplement: S5 Fig — (A) ZnDTC docks onto E. histolytica CSN5. Note the hydrogen bonds (blue lines) formed between ZnDTC drug (yellow) and the metalloprotease site Asp147 and His136. (B) Dose response curve showing increased resistance to ZnDTC treatment by E. histolytica parasites overexpressing CSN5. (C) Infection rate measured by ameba culture of cecal content, n = 7 mice per group. ***P < .001, Fisher’s exact test. (PDF) [file ppat.1008952.s005.pdf]

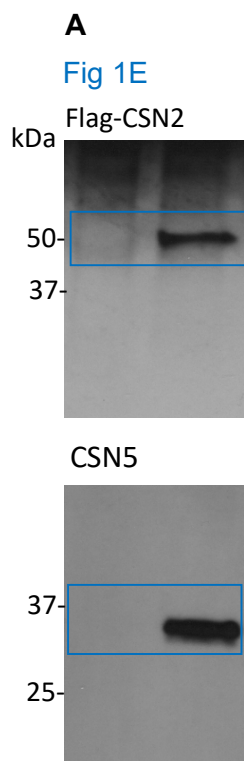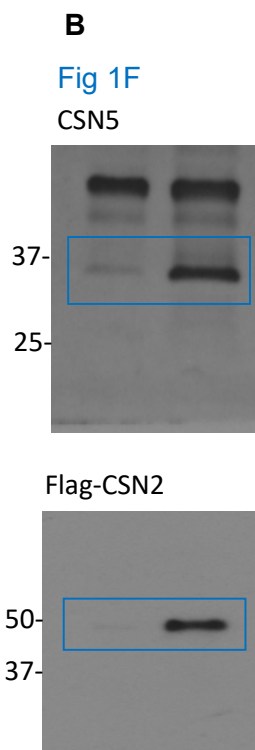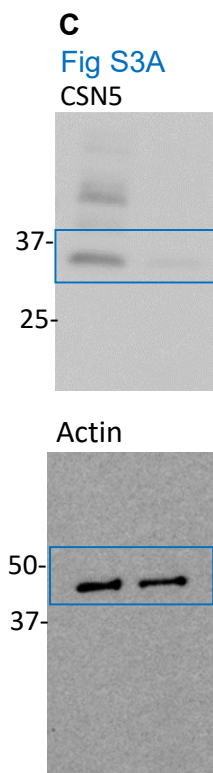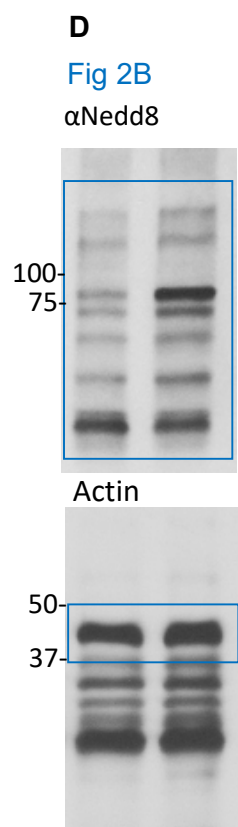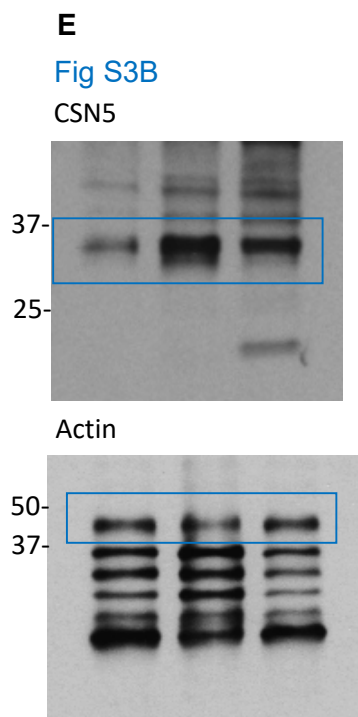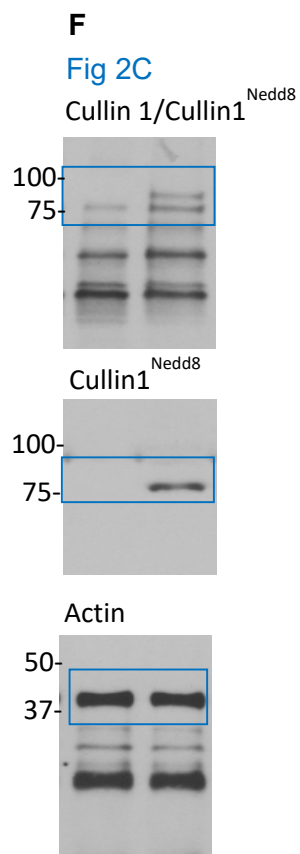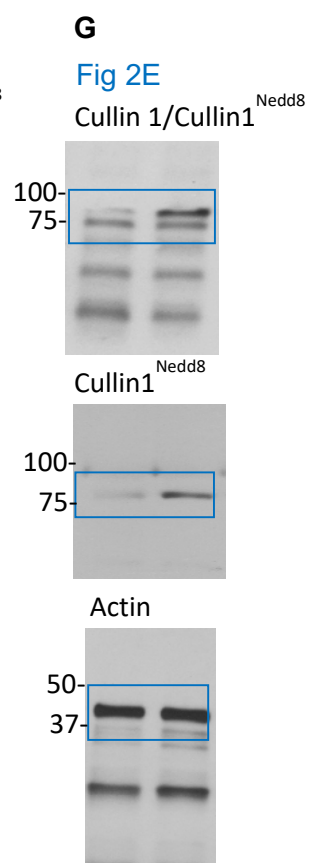

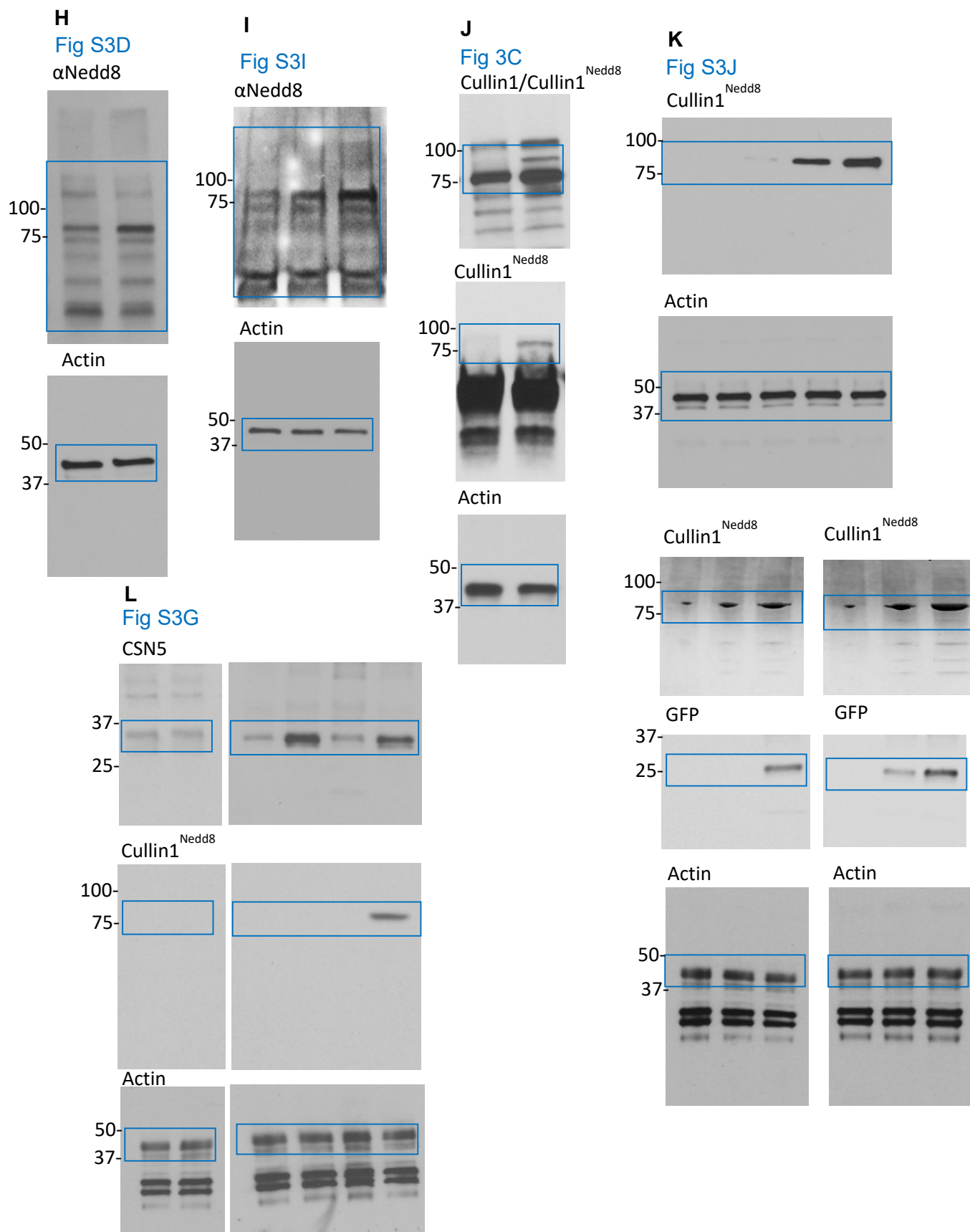

Supplement: S6 Fig — Actin is used as a loading control. Actin levels are similar within the groups that are being compared including blots with actin breakdown products. (PDF) [file ppat.1008952.s006.pdf]
