# Supplementary material for: COP9 signalosome is an essential and druggable parasite target that regulates protein degradation
Source: PLoS Pathog. 2020 Sep 22;16(9):e1008952. doi: 10.1371/journal.ppat.1008952 (PMC7531848; doi:10.1371/journal.ppat.1008952)
Supplement: S2 Fig — (A) Schematic of procedure for identifying protein-protein interaction using co-immunoprecipitation with specific anti-CSN5 or control antibodies followed by mass spectrometric analysis (color highlighted). (B) CSN subunits amino acid sequences. Peptides unique to the CSN subunits identified as the top proteins detected only in the presence of anti-CSN5 and absent with control antibody co-immunoprecipitation. (PDF) [file ppat.1008952.s002.pdf]

A

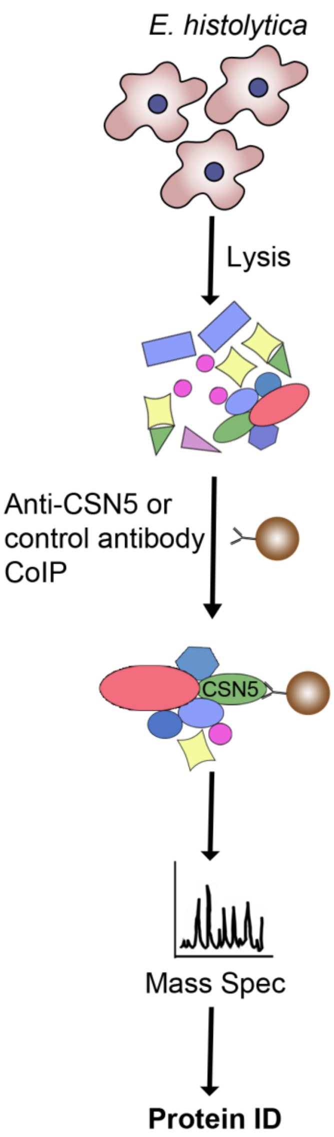

B

CSN5

MGEEAAYKEWEKVNGVKYIEEDK**LLEWNDSER**EQIF**KDRPWKKDPYYFKKCYVSS**
VALLKMVMHAKQGEPLEIMGILIGQTKGDSFVITDVVSLPVEGTETRVNASADCD
AYMLQYGEYKNSTGFKEPFCGWYHSHPSYK**CWLSGIDVATEK**LHQSINDPWIAIV
VDPVTTSTNGKIEIGAFR**TFPEGFKPQQK**AEMKKVLPSEKIADFGSYYDSYYSIK
VELFK**TKLDDQVLR**LLWHEYWINTLAATAIISSRDVMDEKIIDLYDKFTAELKNN
K**SNVVDACGAILDDAKEIQMIYER**GIKSLDLKNILFNQKVTGK

CSN2

MFDDDDIMFDDEGEVIDDEEIGIENKFYDAKNEMETNLEGAIESFK**EIVQEDSEK**
KTEWGYKSLRKLCRYYGKANNEEEFKTYFVQFLEYLNIPAVSKAEKGLFLILGNI
NGMR**NEVVIEVVNK**AIEICEKNSNFSRIIFKLNIKKANTMFESGKYEELKPFLSE
LVNSCYLPNGKEDPMRSHLLIELYGLEIQLYSKLNDMRKLQQLCGKINFSDRNIS
HPKVLGIIMECCGKVK**LCNSDFAGAK**NDFFDSFK**SLDEAGLPER**FDALRFTILAH
LLSSSK**IDIFQAQEVK**SYQRSPEMELVYQLYCAFNENNIIHFKEALNKSGSQFKD
HPYIQQYIPLLIETAQKNLILKLVKCFKRINFSFLAQELDMKEEKVELLVLR**MIF**
DNTLKAKINQFDR**YLIMTEEQSSVTRKYIAITSMSK**TLAASISV

CSN3

MSSSNDLSSVLDK**NQSTIFNEIK**GLK**FSPLTYERYNELLEK**YTISNYPSSHLLIL
KEQTRTLNDKNLSKVISSIK**NYHENISPSMK**KSLEPVFLETLQSVERFCQRQCSL
LDKLTDLVHLLLSEVPDGVLSPYHALLAHILFAKKEYEHGKILYFTKYHKVQQPM
NSFTLQLFLYYSGCIALYNRDLQEAYFLFDQCITTPSK**EITPQCVAAWK**KEALLC
LIINYSLYPVSKQFNYFIDLYQPLLVYK**QIQENFINGPEK**VKFIIEAFSSILK**QD**
GNLGLAKQVVVSYIYSGINKVSK**AFNSISVEALAR**RIHYDKNLLRVGLNKLIKKG
LINASFDGDIIVFEEVKVKDKGLVELLFQLK**ECEQIYEEMK**HHLTIREEEQMNKK
GSIN

CSN1

MMNNYIQSYCKYGRIIRYINLLSNGTIGSQELLEAIDYTELTKNIGGFARLKEAV
SSTLPLDEPEWKKK**IGYSLFEQVKALETEIQSTYGDEER**AKLLYK**LSDIHEEHGE**
YSKAIK**NVLQAVESLK**NK**MMCAEGYYR**LVRLNIFNENFHQATNFLNKLQSLDAFT
GNLYTKFINIISFLLAIRNTETFITAFELLDK**VTTFDEK**DNWEFGEFLSFQDIAI
YGTLIGLLTQK**HQTNVIHLINNSK**FRNHADTVPELVILLDDYKQNKFSK**ICNDVQ**
QLEKYFQFNLYFNQSINNVINCIKRKCYIEYIFAYSVVDMNVMAKMFGDSLMTIE
SALEDYIYSDVIKAKIDAVTHTLNFVDGDER**YHAYESALNAVTKAINLSQEIVLK**
SDAMFDF

CSN6

MTTPEVVLHPLALISITQSATHK**TINCGTNKPER**SVGILLGIENEEEIIVR**TSFE**
IPESQLITKMSEGVK**LNSEVNKEYK**VVGWYAGMANGEPLASDIELHSQIVGENKN
GLFLILNISKCYQKETDKIPITFFVLRNELFVPCSYHIASVDVERIGINEMINAG
SSVETDKK**EKEGISHAIETLKKKVDILVK**YLK**GVENGTIQADNHILAK**IAQICSS
IPVSDNSVFR**EEFNQESNDAK**LTVLMMQLIHTTTVISNNIINYQDLKLQYKNKLN
KEEEERKKAERGMSFDIRRDGVMMAGLEEDYDSDD
