# Supplementary material for: COP9 signalosome is an essential and druggable parasite target that regulates protein degradation
Source: PLoS Pathog. 2020 Sep 22;16(9):e1008952. doi: 10.1371/journal.ppat.1008952 (PMC7531848; doi:10.1371/journal.ppat.1008952)
Supplement: S4 Fig — E. histolytica cullin1 and Nedd8 showing isopeptide bond formation between the conserved lysine (K) residue of cullin1 and the C-terminal glycine (G) of Nedd8. (PDF) [file ppat.1008952.s004.pdf]

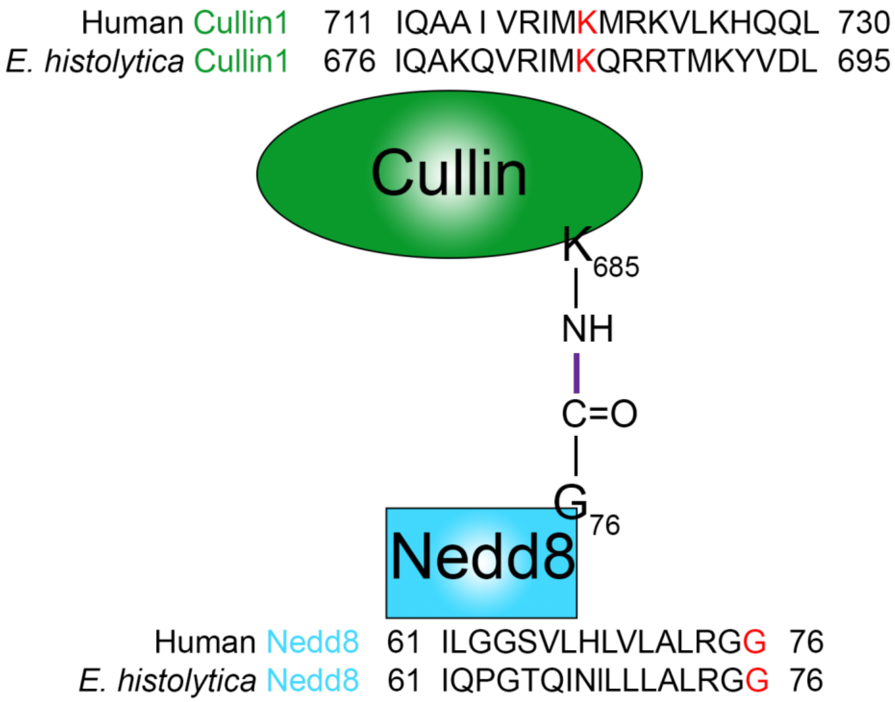

Human Cullin1 711 IQAA I VRIMKMRKVLKHQQL 730
E. histolytica Cullin1 676 IQAKQVRIMKQRRTMKYVDL 695
Cullin
K685
NH
C=O
G76
Nedd8
Human Nedd8 61 ILGGSVLHLVLALRGG 76
E. histolytica Nedd8 61 IQPGTQINILLLALRGG 76
