# Supplementary material for: COP9 signalosome is an essential and druggable parasite target that regulates protein degradation
Source: PLoS Pathog. 2020 Sep 22;16(9):e1008952. doi: 10.1371/journal.ppat.1008952 (PMC7531848; doi:10.1371/journal.ppat.1008952)
Supplement: S2 Table — (PDF) [file ppat.1008952.s008.pdf]

Compound	PubChem ID
1zp5	5326979
8QT	3082386
20(R)-Ginsenoside Rh2	54580480
Actinonin	443600
Alendronate, Na salt	23681107
AM-2	54687474
4-Aminobenzoyl-Gly-Pro-D-Leu-D-Ala hydoxamic acid	16218906
ARP 100	10044321
ARP101	11292680
Apratastat	11452716
AZD1236	24751752
Batimastat	5362422
Capzimin	126599606
CHEMBL196647	44402021
CHEMBL197921	44405997
CHEMBL200385	44405998
CHEMBL251916	9913488
CHEMBL251918	9936194
CHEMBL252117	9892321
CHEMBL254191	9949549
CHEMBL254396	44445389
CHEMBL254397	18379613
CHEMBL254398	18379668
CHEMBL254399	44445390
CHEMBL254603	18379555
CHEMBL254604	9865191
CHEMBL254605	44445391
CHEMBL254606	10971085
CHEMBL254816	44445393
CHEMBL254817	10001964
CHEMBL254818	9866486
CHEMBL255030	9888922
CHEMBL262397	44402280
CHEMBL362651	11261246
CHEMBL398639	44445392
CHEMBL398640	18379569
CHEMBL398641	9869597
CHEMBL400083	9936681
CHEMBL400258	44445388
CHEMBL400463	18379626
CHEMBL41928	44286483

Compound	PubChem ID
CHEMBL428767	4445394
CHEMBL442445	9804305
CHEMBL447211	44591823
CHEMBL449228	25263244
CHEMBL450479	44563467
CHEMBL450549	25263234
CHEMBL452947	44563494
CHEMBL469990	44591880
CHEMBL470830	54726662
CHEMBL470831	54685358
CHEMBL473611	25064051
CHEMBL474456	25065318
CHEMBL474523	44563402
CHEMBL474723	44563403
CHEMBL474924	44563364
CHEMBL474925	44563365
CHEMBL475230	25065946
CHEMBL475619	25263232
CHEMBL482569	44591821
CHEMBL482570	44591824
CHEMBL482961	44561819
CHEMBL482962	44591820
CHEMBL488617	44563487
CHEMBL492314	25263233
CHEMBL492315	25263235
CHEMBL492531	25066563
CHEMBL492599	44563466
CHEMBL492904	25263242
CHEMBL492905	25263243
CHEMBL492953	25263239
CHEMBL493356	25067178
CHEMBL493750	25066564
CHEMBL493958	25263241
CHEMBL494528	25066765
CHEMBL494529	25263237
CHEMBL496909	11602198
CHEMBL496910	44592482
CHEMBL498272	16726064
CHEMBL498274	44592483
CHEMBL499426	44563404
CHEMBL502079	44563468
CHEMBL502674	44563493

Compound	PubChem ID	Compound	PubChem ID
CHEMBL508335	25141117	Marimastat	119031
CHEMBL509270	44563491	Metastat	54678924
CHEMBL510694	44563490	Minocycline	54675783
CHEMBL512856	54726663	MMP Inhibitor II	4218
CHEMBL514336	44563405	MMP Inhibitor III	4219
CHEMBL515439	25263245	MMP Inhibitor V	9888141
CHEMBL518974	44591822	MMP-2 Inhibitor I	6436367
CHEMBL522422	44563469	MMP-2 Inhibitor II	221673413
CHEMBL523001	25067176	MMP-2/MMP-3 Inhibitor I	16760550
CHEMBL523035	25263236	MMP-2/MMP-3 Inhibitor II	3728863
CHEMBL523201	25066763	MMP-2/MMP-9 Inhibitor I	4222
CHEMBL523357	25263238	MMP-2/MMP-9 Inhibitor II	9822095
CHEMBL523670	25066566	MMP-2/MMP-9 Inhibitor V	312659106
CHEMBL523683	25263240	MMP-3 Inhibitor	139211196
CHEMBL523915	44592481	MMP-3 Inhibitor III	9887870
CHEMBL523999	44563465	MMP-3 Inhibitor IV	3728864
CHEMBL525733	16726687	MMP-3 Inhibitor V	4226
CHEMBL609346	44563492	MMP-3 Inhibitor VIII	10549329
Chlorhexidine, dihydrochloride	9571016	MMP-8 Inhibitor I	10761128
CGS27023a	9888897	MMP-9 Inhibitor I	21310926
cis-ACCP	44457233	MMP-9/MMP-13 Inhibitor I	9983251
CL 82198 Hydrochloride	16760373	MMP-9/MMP-13 Inhibitor II	4230
Collagenase Inhibitor 1	11113112	MMP-13 Inhibitor	5289110
CP 471474	9907286	MMP408	25066764
CSN5i-3	129892190	N-Dansyl-D-phenylalanine	13734200
Dimercaptopropanesulfonate	23676755	NNGH	448002
Doxycycline HCl	54685920	NSC 23766	16759159
Doxycycline Hyclate	5486183	o-Phenanthroline monohydrate	21226
Ecotin, Ecoli	7979580	PD166793	9918908
EGTA	6207	Penicillamine	5852
4-epi-Chlortetracycline HCl	54710414	Phosphoramidon	445114
4-epi-demeclocycline	54731359	Prinomastat	466151
Funalenone	312243941	Pro-Leu-Gly hydroxamate HCl	16219864
GM 1489	10322165	Rebimastat	9913881
GM 6001	132519	Ro 31-4724	5487313
H18	1266032	Ro 32-3555	9824350
HJS30LG6HJ	9824010	RS-104966	404615371
HY-114418	9956699	S-3304	10718956
Ilomastat	132519	SB-3CT	9883002
Keracyanin chloride	29231	Tanomastat	6918336
Lactobionic acid	7314	TAPI-0	21881944
Luteolin	5280445		

Compound	PubChem ID
TAPI-1	9827273
TAPI2	134693925
Thiorphan	3132
Trientine hydrochloride	71433
UK 356618	10370504
UK 370106	9808181
WAY033	6852146
WAY 170523	9830392
ZINC00229666	761006
ZINC02860431	2213585
ZINC19438618	24695155
ZINC19476737	19593101
ZINC20231914	*
ZINC22145620	16777008
ZINC33510136	*
ZINC37417840	43244918
ZINC39475452	*
ZINC40403881	25066149
ZINC40897684	25065515
ZINC40914527	25263230
ZINC40914529	25263231
ZINC40914758	25066153
ZINC40914851	25065514
ZINC41498653	*
ZINC57989822	82160883
ZINC62430943	*
ZINC71632004	82307332
ZINC71694581	*
ZINC71726611	*
ZINC76186843	*
ZINC76441060	*
ZINC82305218	*
ZINC82401153	*
ZINC82555302	82480989
ZINC82867972	*
ZINC82973537	82614354
ZINC83306707	*
Zinc ditiocarb	26633
Zinc methacrylate	10130896

S2 Table. Compounds used in screen. Two hundred and five (205) metalloprotease inhibitors from multiple studies that all together screened millions of compounds*. Two compounds dock onto *E. histolytica* CSN5, and formed hydrogen bonds with the metalloprotease site, zinc ditiocarb (ZnDTC) and CSN5i-3.

*References

1. Benjamin, M.M. and R.A. Khalil, Matrix metalloproteinase inhibitors as investigative tools in the pathogenesis and management of vascular disease. Exp Suppl, 2012. 103: p. 209-79.
2. Abbenante, G. and D.P. Fairlie, Protease inhibitors in the clinic. Medicinal Chemistry, 2005. 1(1): p. 71-104.
3. Breuer, E., J. Frant, and R. Reich, Recent non-hydroxamate matrix metalloproteinase inhibitors. Expert Opinion on Therapeutic Patents, 2005. 15(3): p. 253-269.
4. Campestre, C., et al., N-Hydroxyurea as zinc binding group in matrix metalloproteinase inhibition: mode of binding in a complex with MMP-8. Bioorg Med Chem Lett, 2006. 16(1): p. 20-4.
5. Castro, M.M., et al., Matrix metalloproteinase inhibitor properties of tetracyclines: therapeutic potential in cardiovascular diseases. Pharmacol Res, 2011. 64(6): p. 551-60.
6. Catterall, J.B. and T.E. Cawston, Drugs in development: bisphosphonates and metalloproteinase inhibitors. Arthritis Res Ther, 2003. 5(1): p. 12-24.
7. Choi, J.Y. and R. Fuerst, Virtual High-Throughput Screening for Matrix Metalloproteinase Inhibitors. Methods Mol Biol, 2017. 1579: p. 259-271.
8. Hu, J., et al., Matrix metalloproteinase inhibitors as therapy for inflammatory and vascular diseases. Nat Rev Drug Discov, 2007. 6(6): p. 480-98.
9. ncbi.nlm.nih.gov/pccompound/?term=chelating+agents
10. Ledour, G., et al., Introduction of the 4-(4-bromophenyl)benzenesulfonyl group to hydrazide analogs of Ilomastat leads to potent gelatinase B (MMP-9) inhibitors with improved selectivity. Bioorg Med Chem, 2008. 16(18): p. 8745-59.
11. Li, W., et al., A selective matrix metalloprotease 12 inhibitor for potential treatment of chronic obstructive pulmonary disease (COPD): discovery of (S)-2-(8-(methoxycarbonylamino)dibenzo[b,d]furan-3-sulfonamido)-3-methylbutanoic acid (MMP408). J Med Chem, 2009. 52(7): p. 1799-802.
12. Li, K., F.R. Tay, and C.K.Y. Yiu, The past, present and future perspectives of matrix metalloproteinase inhibitors. Pharmacol Ther, 2020. 207: p. 107465.
13. Li, J., et al., Capzimin is a potent and specific inhibitor of proteasome isopeptidase Rpn11. Nat Chem Biol, 2017. 13(5): p. 486-493.
14. scbt.com/browse/chemicals-Protein-Interacting-Inhibitors-Activators-Substrates-Protein-Inhibitors-MMP-Inhibitors/_/N-169lucc
15. V, M., et al., Matrix metalloproteinase protein inhibitors: highlighting a new beginning for metalloproteinases in medicine. Metalloproteinases In Medicine, 2016. 2016(3): p. 31-47.
16. Raffetto, J.D. and R.A. Khalil, Matrix metalloproteinases and their inhibitors in vascular remodeling and vascular disease. Biochem Pharmacol, 2008. 75(2): p. 346-59.
17. Rossello, A., et al., A new development of matrix metalloproteinase inhibitors: twin hydroxamic acids as potent inhibitors of MMPs. Bioorg Med Chem Lett, 2005. 15(9): p. 2311-4.
18. Schlierf, A., et al., Targeted inhibition of the COP9 signalosome for treatment of cancer. Nat Commun, 2016. 7: p. 13166.
19. Sterling, T. and J.J. Irwin, ZINC 15--Ligand Discovery for Everyone. J Chem Inf Model,

2015. 55(11): p. 2324-37.
20. Vandenbroucke, R.E. and C. Libert, Is there new hope for therapeutic matrix metalloproteinase inhibition? Nat Rev Drug Discov, 2014. 13(12): p. 904-27.
21. Whitlock, G.A., et al., A novel series of highly selective inhibitors of MMP-3. Bioorg Med Chem Lett, 2007. 17(24): p. 6750-3.
22. Yan, Y.L., et al., Synthesis of hydroxypyrone- and hydroxythiopyrone-based matrix metalloproteinase inhibitors: developing a structure-activity relationship. Bioorg Med Chem Lett, 2009. 19(7): p. 1970-6.
